# Prescribing habits and caregiver satisfaction with resources for dosing children: Rationale for more informative dosing guidance

**DOI:** 10.1186/1471-2431-11-25

**Published:** 2011-04-02

**Authors:** Jeffrey S Barrett, Mahesh Narayan, Dimple Patel, Athena F Zuppa, Peter C Adamson

**Affiliations:** 1 Department of Pediatrics, Division of Clinical Pharmacology and Therapeutics, The Children's Hospital of Philadelphia, 3501 Civic Center Blvd, Philadelphia, PA, 19104, USA

**Keywords:** pharmacotherapy guidance, caregiver role, patient individualization, pediatric prescribing habits

## Abstract

**Background:**

Physicians, nurses and hospital pharmacists were surveyed to assess attitudes of hospital-based pediatric caregivers regarding the dosing of medicine to children. Our objectives were to gauge how current resources are utilized to guide the management of pediatric pharmacotherapy, assess drugs and drug classes where guidance is most critical and examine the prevalence and practice of dose adjustment in pediatric patients.

**Methods:**

Questionnaire categories included demographics, pharmacotherapy resources, dosing adjustment and modification, and valuation of additional tools to provide improved pharmacotherapy guidance. The questionnaire was developed in collaboration with representative nurse, pharmacist and physician team members using the SurveyMonkey.com site and survey tool. The survey link was distributed to caregivers via email. The questionnaire results of 303 respondents were collected into MS Excel and imported into SAS for data summarization.

**Results:**

A total of 313 responses were obtained. Physician and nurse practitioner groups comprised the majority of the responses. Approximately 80% of the responders considered dosing adjustment important in pediatric pharmacotherapy. While there was general satisfaction with available resources, nearly 75% responded in support of access to predictive tools that facilitate individualized patient pharmacotherapy. The majority of respondents (> 65%) indicated that dosing outside standard practice occurs in 1-20% of their patients, while still a substantial number of respondents (a range of 8 to 20% reflecting the resident and fellow categories) estimated between 20 and 50% of their patients required adjustments outside the standard practice.

**Conclusions:**

Differences in prescribing habits based on caregiver role, specialty and location were small and likely require further exploration. Existing resources are generally viewed as helpful but inadequate to guide recommendations for individual patients. Decision support systems connected to hospital-based electronic medical records offer the promise of informative and individualized pharmacotherapy guidance.

## Background

Children represent a dynamic target for prescribing pharmacotherapy as age, size, organ function and developmental state are factors that contribute to the variation in drug response that limit the simplistic scale-down from the adult "one size fits all" dosing approach[1]. While this concept is reasonably well appreciated by pediatric caregivers, the extent to which this appreciation translates into rationale dosing guidance in children is unknown. Likewise, while prescribing to pediatric in-patients is decidedly focused on the individual patient with respect to dosing, this desire is often in conflict with data generated by the drug sponsor where the resultant prescribing information emphasizes the average or typical patient, providing guidance to aggregate "special" populations. The introduction of new agents on formulary may provide an improvement in clinical options but often further complicates prescribing practice. In addition, prescribing patterns change constantly and are not entirely generalizable across institutions[2]. Many studies support the correlation between deficient drug prescribing and poor adherence to evidence based treatment guidelines, inadequate individual dosage adjustments and adverse drug events [3-5].

Caregiver role is an important factor in the definition of such patterns within an institution. While there is a general workflow of order, verification and review shared by the physician, nurse and pharmacist, the specific checks and balances put in place to ensure accurate prescribing and administration in an in-patient setting are often unique to the subspeciality [6]. Johnson et al [7] have previously examined the incidence of discrepancies among written prescriptions, medication regimens and patient discharge instructions sheets and the actual labels on medications dispensed by community pharmacies. The study documented prescriber errors in dosing frequencies and formulations in addition to altered prescriptions by the community pharmacists. The authors called for improved education and risk management efforts encouraging caregivers to consult appropriate reference materials to ensure that dose formulations and guidelines are accurate.

In fact, resources available to guide pediatric dosing are few and the lack of resources well appreciated [8-10]. The most commonly appreciated resources include the drug monograph or label (package insert) available in paper and electronic forms and captured in compendia guides such as the physician's desk reference (PDR). The source studies described in the package insert are typically limited to those conducted by or on behalf of the drug sponsor but may also include literature studies summarized by the drug sponsor. As the drug sponsor must petition the FDA to include the proposed material in the package insert, not all of the available information is included in the drug monograph. Other compendia sources such as the Lexi-Comp (http://www.lexi.com/) or other pediatric dosing handbooks such as Harriet Lane[11] attempt to review the relevant literature and provide periodic updates. These are likely the best reflection of current information regarding dosing guidance in pediatrics. However, there is often little interpretation and it is challenging to synthesize the body of small discrete studies into a meaningful prescribing practice particularly when the source studies are conducted for regulatory purposes and not for informative dosing guidance. More importantly, the format of this information is static and text based. While on-line versions of Lexi-Comp and other tools have made marked improvements with respect to access and retrieval, it is still not in the scope of the resource to interpolate, extrapolate or otherwise summarize the information provided except through the interpretation of the reader.

We have previously studied drug utilization patterns in the pediatric ICU[12], developed visualization tools to mine and query utilization patterns in the hospital in-patient setting[13], developed a key performance index (KPI) scoring system to rank and prioritize agents on formulary for future study[14], and described how predictive models can inform decision support system that interface with the hospital's electronic medical records (EMRs)[15]. Our objective for this investigation was to assess pediatric caregiver prescribing habits, including attitudes with respect to their valuation of available resources to guide pediatric pharmacotherapy. We were also interested in their opinions on dosing adjustments specifically in the identification of agents difficult to manage, the frequency of dosing modifications (beyond the standard of care) in their practice and the factors they deem as critical criteria to guide such adjustments. The results of the 15-question survey were analyzed across caregiver role and serve as the baseline assessment for the development of decision support systems that will serve as a future, dynamic resource to guide pediatric pharmacotherapy with emphasis on individualized recommendations and personalized, safe drug therapy.

## Methods

### Clinical Setting

The medical staff at The Children's Hospital of Philadelphia (CHOP) includes approximately 900 Attending Physicians, 223 Physician Fellows, 135 Physician Residents and 1900 Nurse Practitioners. These staff members all have input into the prescribing decisions made at CHOP. Additionally, there are 45 hospital pharmacists on staff with pharmacists having specialized roles (clinical specialists) within therapeutic areas. Pharmacy responds to drug information inquires ranging from drug, dosage or dosage form recommendations to extensive literature searches on specific pharmacotherapeutic topics. The pharmacy service reviews all therapeutically monitored drug concentrations reported by the clinical laboratory twice daily. Medical staff is contacted with recommendations if dosage adjustments are required. Pharmacokinetics consultations are also provided upon request of the medical staff.

The protocol for this investigation was approved by the Institutional review Board of The Children's Hospital of Philadelphia. A waiver of HIPAA authorization under 45 CFR 165.512(i)(2)(ii) was granted based on the nature of the study evaluation. A waiver of assent and parental permission and consent was also granted because the study met the criteria under CFR 46.116(d), due to its de-identified and retrospective design.

### Questionnaire

A 15 question survey was prepared based on the feedback from a pilot questionnaire and specific comments from each of the target caregiver roles (physician, nurse and pharmacist). The pilot survey targeted approximately 30 pediatric caregivers and following interviews with the questionnaire respondents, refined to the final questionnaire. The final questionnaire was composed of six tick-box questions, seven 3-4 point scale responses (seven of which allowed comments) and 2 free text questions; the actual questions and response options are provided in the Appendix (see Additional file 1).

The survey of attending physicians, fellows, residents, nurse practitioners, clinical pharmacists, physician assistants and clinical nurse specialists was distributed through the Survey Monkey (Portland, Oregon USA; http://www.surveymonkey.com/) web-application via internal email to approximately 900 pediatric caregivers within the institution (926 was the actual number of email recipients). The 4 domains surveyed included demographics, pharmacotherapy resources, dosing adjustment and modification, and valuation of additional tools to provide improved pharmacotherapy guidance. Questions considered the pediatric caregiver's role, specialty and location, as well assessing prescriber knowledge regarding dosing guidance and attitudes toward dose modification and patient individualization. The survey also focused on accessibility, ease of use and appropriateness of existing resources regarding pediatric dosing guidance. Information regarding the frequency of dosing modification along with consultation of dosing compendiums and estimation of success rate in dosing guidance was acquired. The sampled population of caregivers was largely based on the availability of mailing lists in which the caregiver role could be assured. The greater representation of physicians from the in-patient setting likewise reflects the fact that this population is collectively identified by group lists within the institution.

The responses were imported into SAS for further summarization and analysis. Missing data values were excluded from the frequency counts.

## Results

### Demographics

**Figure 1:**
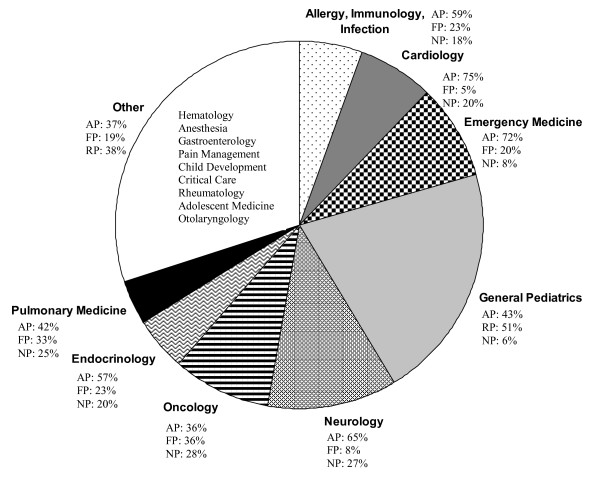
**Specialization of pediatric caregivers participating in a survey on valuation of pharmacotherapy resources and pediatric prescribing habits at the Children's Hospital of Philadelphia (n = 303)**. AP: Attending Physicians; FP: Physician Fellows; RP: Resident Physicians; NP: Nurse Practitioners

Surveys were distributed via email and up to 4 reminders were issued over a 3 week period. A total of 313 completed surveys were received from the 926 targeted caregivers. The 34% response rate does not reflect an adjusted rate[16] based on acknowledged email receipt and likely under-estimates the actual response which is likely greater than 40% based on typical overestimation of the denominator for email-based surveys[17]. Post hoc analysis revealed that several caregivers within each of the 4 email group lists targeted with either incorrectly assigned or no longer at the institution; exact counts were not confirmed. The distribution of caregiver roles included 151 (48% of the total response) attending physicians, 69 (22%) nurse practitioners, 46 (15%) fellows, 37 (12%) residents, 6 (2%) clinical pharmacists, 3 (1%) physician assistants and 1 (0.3%) clinical nurse specialist. The last three categories were excluded from the analysis summary due to the low response frequency (10 responses in total) leaving an evaluable dataset of 303 responses. Within the top five specializations, 65 were from General Pediatrics, 37 from Neurology, 27 from Oncology, 25 from Emergency Medicine and 22 from Cardiology. As expected, the response rate for these specialties is correlated with their size. Figure 1 shows the intersection of caregiver role and clinical specialty from our surveyed population. Regarding location, 125 (83%) of the attending physicians were located on the main campus, 13 (9%) in specialty care centers and 12 (8%) in primary care centers. In the nurse practitioner category, 57 (83%) were located on the main campus, 7 (10%) in specialty care centers and 3 (4%) in primary care centers. For fellows, 45 (98%) were located in the main campus setting and 1 (2%) in primary care centers. All 37 residents were located on the main campus.

### Pharmacotherapy resources

The most common drug information resources currently available to the pediatric caregiver are summarized in Table 1. With respect to the value attributed to the existing resources, attending physicians and nurse practitioners were split between 'very' and 'somewhat' informative while 63% of the fellows and 76% of the residents described the available resources as 'very informative.' Relatively few caregivers found the available resources to be 'not very informative.' Based on the survey response, attending physicians preferred the online Lexi-Comp system (77.5%), followed by past experience (59%) and consultation with the hospital pharmacist (53%). Fellows favored Lexi-Comp online (87%), followed by hospital pharmacist consultation (54%) and past experience (33%). Residents overwhelmingly preferred Lexi-Comp online (92%) but also Sunrise Clinical Manager (51%) followed by the hospital pharmacist (46%). Nurse practitioners preferred Lexi-Comp Online (83%) as well, followed by the hospital pharmacist (45%) and past experience (45%). Differences between physician classes likely reflect prescribing frequency and experience as well as comfort with information technology.

**Table 1:** Caregiver-identified preferences of sources for pediatric dosing guidance

Count (% of total in clinical role category)										
**Clinical Roles**	**Physician's Desk Reference**	**Lexi-Comp Handbook**	**Harriet Lane Handbook**	**Sunrise Clinical Manager**	**Lexi-Comp Online**	**Scientific Literature**	**Hospital Pharmacist**	**Past Experience**	**Epocrates**	**Other**

Attending Physicians	42 (27.8)	43 (28.5)	44 (29.1)	31 (20.5)	117 (77.5)	62 (41.1)	80 (53)	89 (58.9)	19 (12.6)	39 (25.8)

Physician Fellows	4 (8.7)	5 (10.9)	13 (28.3)	10 (21.7)	40 (87.0)	10 (21.7)	25 (54.3)	15 (32.6)	9 (19.6)	12 (26.1)

Resident Physicians	-	7 (18.9)	10 (27.0)	19 (51.4)	34 (91.9)	3 (8.1)	17 (45.9)	6 (16.2)	5 (13.5)	6 (16.2)

Nurse Practitioners	21 (30.4)	23 (33.3)	9 (13.0)	13 (18.8)	57 (82.6)	5 (7.2)	31 (44.9)	31 (44.9)	6 (8.79)	15 (21.7)

(Multiple selections permitted)

Glossary:

**Physician's Desk Reference**: commercially published compilation of prescribing information on prescription drugs, updated annually.

**Lexi-Comp**: a pediatric-specific reference for pharmacotherapy guidance and drug formulary information (available in hardcopy and electronic formats; CHOP has on-line version accessible from EMR system).

**Harriet Lane Handbook**: Reference for pediatric diagnostic and management guidance, recommended tests, therapeutic information, and comprehensive drug formulary.

**Sunrise Clinical Manager**: an on-line patient data and lab ordering system.

**Epocrates**: medical software for drug interaction, drug prices, drug dosing and disease management

### Dosing adjustment and modification

Table 2 summarizes questionnaire responses that examined the attitudes of caregivers regarding dose adjustment, the frequency they access existing resources and the convenience and value they place on the guidance they abstract from these resources. It is clear that resources to support dosing guidance are viewed as valuable. Approximately 80% of the respondents stated that checking more than one reference source occurs less than 25% of the time while 8 (residents) to 20% (fellows) responded that this occurs between 25 and 50% of the time. Not surprisingly, the majority (> 75%) of pediatric caregivers rate dose adjustment as being '*very important*' with more than 20 rating it as '*somewhat important*'. Regarding the convenience of obtaining dosing guidance, most responded that the availability was either somewhat or very convenient. The majority of respondents indicated that dosing outside standard practice occurred in 1-20% of their patients, while still a substantial number of respondents (a range of 8 to 20% reflecting the resident and fellow categories)estimated between 20 and 50% of their patients required adjustments outside the standard practice. There was some difference in the response rates by caregiver role for this question, particularly between fellows and residents.

**Table 2:** Prescribing practice, valuation of dose adjustment and pharmacotherapy guidance by caregiver role

		% Response (within role)				
**Question**	**Response**	**Attending Physician**	**Physician Fellow**	**Resident Physician**	**Nurse Practitioner**	**Overall**

Frequency of checking more than one source to obtaining dosing guidance	Never	6	2.2	8.1	5.8	5.3
	
	<25% of the time	79.4	78.2	81.1	78.2	**79.5**
	
	25-50% of the time	12.6	19.6	8.1	14.5	13.6
	
	>50% of the time	2	0	2.7	1.5	1.7

Value of dosing adjustments in pediatrics	Not very important	2.7	0	0	1.4	1.7

	Somewhat Important	20.5	17.4	24.3	23.2	21.1
	
	Very Important	76.8	82.6	75.7	75.4	**77.2**

Convenience of information on dosing guidance	Not very Convenient	7.9	8.7	2.7	8.7	7.6
	
	Somewhat Convenient	60.9	71.7	43.2	50.7	**58.1**
	
	Very Convenient	31.3	19.6	54.1	40.6	34.3

Frequency of patients requiring modification outside "standard" dose recommendations	<1% of patients	14	4.3	18.9	20.6	14.6

	1-20% of patients	66.7	73.9	73	52.9	**65.4**
	
	20-50% of patients	12.7	19.6	8.1	16.2	14.0
	
	>50% of patients	6	2.2	0	7.4	5.0
	
	Other	0.7	0	0	2.9	1.0

Value of tools for individualized dosing guidance	Yes	69.5	80.4	78.4	68.2	**72.0**

	No	3.3	0	5.4	1.4	2.6
	
	Maybe	27.2	19.6	16.2	30.4	25.4

(highest overall response per question in bold) (Response assessed as % category within caregiver role)

### Valuation of additional prescribing tools

The value of tools that would provide individualized dosing guidance was strongly endorsed by the questionnaire response with over 70% stating that these would be desirable. Lack of user friendliness, error-proof guarantee and information on drug metabolism and pharmacokinetics-pharmacodynamics (PK-PD) were the highest cited drawbacks of respondents. The next highest cited complaints were inconsistent information, too population-centric and too patient-centric at 15, 4 and 2% of the total responses. Approximately 20% of those surveyed felt that there were no drawbacks.

Dose modification outside the standard dose requirements while occurring infrequently (50-70% of the pediatric caregivers modified dosages in only '1-20% of patients') reflects individual patient factors. As expected, weight, organ function and age top the list of factors described as critical by questionnaire respondents (Appendix, Question 8, see Additional file 1). Body-surface area and height were also cited although to a lesser extent; these responses likely reflect specific drugs and classes. Table 3 summarizes drug classes or indications identified as difficult to manage by clinical specialty/setting. Overall, antibiotics (21%) are viewed as the most complicated to manage, followed by anticonvulsants (18%) and anticoagulants (15.5%). The proximity of these responses suggests that there is no real difference among caregivers between these drug classes.

**Table 3:** Medication classes identified as difficult to manage+ (303 evaluable respondents*)

Rank	Classes/Agents Cited within Specialty					
	
	Allergy and Immunology; Infectious Disease	Cardiology	Emergency Medicine	General Pediatrics	Neurology	Oncology
1	Antibiotic, Antifungal	Anticoagulant	Antibiotics, Anticonvulsant, Antiemetic	Anticonvulsant	Anticonvulsant	Antineoplastic

2	Antiviral	Antiarrhythmic	Antianxiety, Antiarrhythmic, CNS Agents	Antibiotic	Antibiotics, Anticoagulant	Anticonvulsant, Orphan Drug

3	Anti-infective, Asthma	Antihypertensive, Immunosuppressant	Antidepressants, Immunosuppressant	ADHD	Antiarrhythmic, CNS Agents	Anticoagulant, Antifungal

4	Antihistamine	Antibiotic	ADHD, Antihypertensive, Antineoplastic	Antidepressant, CNS Agents	Antihypertensive	Antianxiety, Antidepressant

5	AIDS, Immunosuppressant	Anti-anxiety, Anticonvulsant, CNS Agents, Orphan Drugs	Anticoagulant, Antifungal, Anti-infective, Orphan Drugs	Anticoagulant, Antifungal	Antifungal, Orphan Drugs	ADHD

* Note: Responses pooled across caregiver role

+ Tied ranks listed based on equivalent respondent counts

## Discussion

The results of this survey confirm the importance of dosing guidance for the management of pediatric pharmacotherapy among various caregiver roles and specializations. They also confirm the necessity of getting feedback from this diverse community as there are differences of opinion that can influence the acceptance of new information and approaches as well as the implementation of new technology which offers the potential to improve outcomes. This initial assessment was designed to serve as a baseline response from the caregiver community prior to the development, assessment and hopefully future implementation of a pediatric knowledgebase that provides real-time, individualized guidance for dosing and managing drug therapy in children.

Some obvious trends appear to reflect the seniority of the caregiver. Specifically, the value placed on the scientific literature within the physician community would seemingly correlate with age and experience with 41.1, 21.7 and 8.1% of attendings, fellows and residents respectively responding that they refer to the scientific literature for dosing guidance. It may also reflect the time that each of these roles has to devote to searching and reviewing the literature. Likewise, it is not surprising that only 16% of the residents cite 'past experience' as a resource for pharmacotherapy guidance. Perhaps consistent with their generation, residents would seemingly be more comfortable with information technology as 92% refer to Lexi-Comp Online and 51% use Sunrise Clinical Manager (the EMR system; as opposed to ~20% for the other responders).

Compared to 51% of the attending physician community and 48% of the nurse practitioners, over 75% of the residents categorize compendial information to be "very informative." It may also suggest that the surveyed attending physicians and nurse practitioners are generally more experienced and hence less dependent on such compendiums. Residents and nurse practitioners have similar responses throughout which may be due to the fact that they are responsible for most of the actual ordering in the hospital. It is interesting to note that 81% of the residents use SCM compared to attendings (33%), fellows (39%) and nurse practitioners (30%). As residents are extensively engaged in ordering and prescribing, which is primarily accomplished through SCM at the moment, this is also not surprising. It was somewhat surprising that antibiotics were identified as a difficult to manage drug class given that there is generally more data/experience with this class than others. This likely reflects the diversity in specialty and experience as well.

Hence, age, experience, specialty and role of the pediatric caregiver appear to be key factors underlying differences in how individual caregivers respond to clinical decisions regarding dosing children as well as educate themselves with available resources to further guide them[18]. While technologic advances such as Computerized Physician Order Entry (CPOE) systems have the potential to greatly reduce human error, their actual performance is highly variable[19,20]. It has been maintained that the strategy for preventing errors and adverse events in health care must involve tools that can improve communication, make knowledge more readily accessible, require key pieces of information, assist with calculations, perform checks in real-time, assist with monitoring and provide decision support[21]. This functionality is currently unavailable at many pediatric in-patient centers. Hence, satisfaction with existing resources should not prevent the construction and deployment of tools that enhance patient safety and provide confidence to caregivers with respect to managing their patient's drug therapy. Most importantly, it is clear that this community must be continually engaged to ensure that new technology is properly scrutinized and evaluated prior to and during implementation.

Given the diversity in experience and specialization of the pediatric caregiver community, it seems obvious that drug and disease-specific guidance with reference to the individual patient would facilitate more standard practices around dosing adjustments and raise the overall knowledge on pediatric clinical pharmacology and therapeutics. This is especially relevant given the concerns about the adequacy of training in pediatric clinical pharmacology and toxicology[5,22-25]. The prevalence of EMR systems among our various pediatric in-patient and out-patient facilities would seem to be a perfect conduit for this information[26] although the task and scope for such a medical informatics system is, as yet, in its infancy. It is clear that the successful development and support for such a system will have to be shared among the various stakeholders and accommodate the requirements from a diverse caregiver community.

These results indicate several limitations with the survey which must be appreciated. First and foremost, it is based on a single institution and the generalizability of these results must consider potential regional differences in prescribing practices as well as differences due to setting (i.e., smaller community-based institutions). Secondly, while efforts were made to ensure a balanced response with respect to caregiver roles and location, we were somewhat limited by the availability of mailing lists that could accurately identify roles as well as caregivers in specialty centers. These were not easily assembled at the time of the survey due, in part, to an antiquated email system that has since been replaced. Finally, the categorical responses defined in the survey questions, while based on the expert opinion of our design group (and reflecting the caregiver community) seemingly lacks the granularity to provide more quantitative point estimates for certain questions. Despite these limitations, we feel the results are robust certainly for our institution and similar large, teaching hospitals in which the care of children is the primary emphasis.

Sjoborg[27] previously reported pilot results from a computerized prescribing system that provides pharmacological knowledge at the point of care. Their approach focused on providing recommendations, alerts for interaction, drug therapy during pregnancy and breast feeding and a search tool for adverse effects through a single database interfaced to their hospital's EMR system. Recognizing the time limitations often presented to our pediatric caregivers, their results would seem to support the proof of concept for this approach. Most importantly, the authors call for a more coordinated effort within and across countries as opposed to the home grown efforts at various academic medical centers[15,28]. Our results would seem to support this finding and suggest further that more dynamic integration of decision analytics to hospital EMRs will also enhance such a knowledgebase[15]. It is also clear that the involvement of the varied caregivers involved in managing drug therapy to children will be essential to ensure that differences in role, specialty, and function are accommodated in both the design and testing of such systems and tools.

## Conclusions

Deriving optimal dosing guidance for children continues to be a concern for pediatric caregivers[29]. The amount of information available for dosing guidance in children, while still inadequate, is more vast and complex than in the past[8]. With the necessity of modifying dose based on age, weight, developmental status, organ function, drug interaction potential and other disease-modifying conditions looming, integrated solutions that synthesize this information should provide more informed decision making. In the past there may have been concerns with physician willingness to trust and utilize such systems[30]. With the continued exposure to information technologies, it is obvious that these concerns are lessening and will eventually be irrelevant[31]. As others have pointed out, the support of new technologies by enlightened leadership will be a critical aspect in the transition to new technologies. It is clear that being satisfied with the status quo benefits neither the quality of clinical decision making nor the care of our patients. Additional resources to guide pediatric pharmacotherapy are needed now and must be based on the currently available knowledge regarding the drug-disease-population interface.

## Competing interests

Financial competing interests

• In the past five years we have not received reimbursements, fees, funding, or salary from an organization that may in any way gain or lose financially from the publication of this manuscript, either now or in the future.

• We do not hold any stocks or shares in an organization that may in any way gain or lose financially from the publication of this manuscript, either now or in the future.

• We do not hold or have applied for any patents relating to the content of the manuscript. We have not received reimbursements, fees, funding, or salary from an organization that holds or has applied for patents relating to the content of the manuscript.

• We do not have any other financial competing interests.

Non-financial competing interests

There are no non-financial competing interests (political, personal, religious, ideological, academic, intellectual, commercial or any other) to declare in relation to this manuscript.

## Authors' contributions

JB, AZ and PA prepared the 15 survey questions in an open ended and close ended form. MN developed the questionnaire using Survey Monkey tool and distributed the survey web link to caregivers via email. MN and DP summarized and performed the statistical analysis of survey results data using SAS. All authors read and approved the final manuscript.

## Authors' information

Dr. Jeffrey S. Barrett is a Research Associate Professor of Pediatrics, University of Pennsylvania, the Director of the Laboratory for Applied PK/PD in the Division of Clinical Pharmacology and Therapeutics at the Children's Hospital of Philadelphia and an Associate Scholar in the Center for Clinical Epidemiology and Biostatistics at The University of Pennsylvania. Dr. Barrett serves as the Principal Investigator for CHOP's Pediatric Pharmacology Research Unit and heads the Kinetic Modeling and Simulation core of the Penn/CHOP Clinical and Translation Science Award. Dr. Barrett's research interest is focused on investigating sources of variation in pharmacokinetics and pharmacodynamics applying clinical pharmacologic investigation coupled with modeling and simulation strategies to pursue rational dosing guidance. He develops pharmacometric approaches to advance PK/PD, medical informatics and disease progression modeling. Dr. Barrett has also integrated model-based decision support systems with hospital electronic medical records and has pioneered the pediatric knowledgebase development program for the past 6 years.

## Source of Funding

This research was supported in part by the Pediatric Pharmacology Research Unit (PPRU) grant, NIH U10, HD037255-06 and the challenge grant, 1RC1LM010367-01, Decision Support System to Guide Pediatric Pharmacotherapy.

## Pre-publication history

The pre-publication history for this paper can be accessed here:

http://www.biomedcentral.com/1471-2431/11/25/prepub

## Supplementary Material

Additional file 1 **Actual Questionnaire - APPENDIX**. • Actual Questionnaire - APPENDIX • PDF (Adobe Acrobat) • Managing Pharmacotherapy in Children There were 15 survey questions designed to assess prescriber's knowledge at The Children's Hospital of Philadelphia regarding dosing guidance and dose modification to identify the problems with pediatric pharmacotherapy today. Click here for file
