# Supplementary material for: Prescribing habits and caregiver satisfaction with resources for dosing children: Rationale for more informative dosing guidance
Source: BMC Pediatr. 2011 Apr 2;11:25. doi: 10.1186/1471-2431-11-25 (PMC3076266; doi:10.1186/1471-2431-11-25)
Supplement: Additional file 1 — Actual Questionnaire - APPENDIX. • Actual Questionnaire - APPENDIX • PDF (Adobe Acrobat) • Managing Pharmacotherapy in Children There were 15 survey questions designed to assess prescriber's knowledge at The Children's Hospital of Philadelphia regarding dosing guidance and dose modification to identify the problems with pediatric pharmacotherapy today. [file 1471-2431-11-25-S1.PDF]

APPENDIX

The listings and summary of the survey responses can be found on our website at http://www.research.chop.edu/programs/cpt/lapkpd/ptherapy.php. The actual questionnaire appears below.

Managing Pharmacotherapy in Children

The following questionnaire was prepared by the Division of Clinical Pharmacology & Therapeutics at CHOP. It is geared towards assessing prescriber knowledge regarding dosing guidance and attitudes toward dose modification and patient individualization. It focuses on accessibility, ease of use and appropriateness of existing data regarding dosing guidance. The results of this survey will help us identify (and quantify) the problems associated with pediatric pharmacotherapy today and will help us fix it for a better tomorrow. We appreciate your time and effort in completing this brief survey.

1. What is your clinical practice role?

□ Attending Physician □ Resident Physician □ Fellow Physician □ Clinical Pharmacist □ Physician Assistant □ Nurse Practitioner □ Other (please specify)

2. What is your area of specialization (e.g., neonatology, oncology, etc)

3. Where do you spend majority of your time within the CHOP system?

□ Main Campus □ Primary Care Center □ Kids' First Network □ Specialty Care Center

4. Where do you currently get reference information for dosing when prescribing a medication? (check all that apply)

□ Physician's Desk Reference (PDR) or Drug Monographs □ LEXI-COMP Handbook □ Harriet Lane Handbook □ Sunrise Clinical Manager □ LEXI-COMP Online □ Clinical Pharmacology Database □ Epocrates Online Premium □Facts and Comparisons 4.0 □ Micromedex □ Epocrates Online Free RxList.com □ Scientific Literature □ Hospital Pharmacist □ Past Experience □ Other Compendium (please specify)

5. How informative are the existing dosing compendiums?

□ Not very informative □ Somewhat informative □ Very informative

6. How often do you have to check more than one source to obtain the dosing guidance you require?

□ Never □ Less than 25% of the time □ Between 25-50% of the time □ More than 50% of the time

7. How important is dose adjustment in the care of your patients?

□ Not very important □ Somewhat important □Very important

8. Rank the following criteria (in terms of priority) upon which you would adjust adult doses for your pediatric patient.

*High Medium *High Medium Low *Low *N/A

*High Medium *High Medium Low *Low *N/A

□ Age □ Body weight □ Height □ Body surface area □ Organ function (i.e., renal, hepatic) □ Other (please specify along with level of priority)

9. How convenient is it for you to obtain information pertaining to dosing guidance?

□ Not very convenient □ Somewhat convenient □ Very convenient

10. What percentage of your patients do you modify dosages outside the standard dose requirements?

□ Less than 1% of patients □ Between 1-20% of patients □ Between 20-50% of patients □ More than 50% of patients □ Other (please specify)

11. Do you use any of the following tools to calculate dose requirements? (check all that apply)

□ LEXI-COMP Online □ SCM □ NeoFax □ Calculator □ Others (please specify)

12. If you do use the aforementioned tools, what are its drawbacks, if any?(check all that apply and please specify the tool)

□ Not user friendly □ Not interactive / modifiable □ Information is variable and inconsistent with other sources □ Not error-proof □ Too patient-centric □ Too population-centric □ No information on drug metabolism □ No information of PK/PD □ No drawbacks
(Please Specify the tool)

13. What medication classes are the most difficult to manage (i.e., where dosing deviates from the recommended dose?). Please check all that apply.

□ ADHD □ AIDS □ Antianxiety □ Antiarrhythmic □ Antibiotics □ Anticoagulant □ Anticonvulsant □ Antidepressants □ Antiemetic □ Antifungal □ Antiinfectives □ Antihistamine □ Antihypertensive □ Antineoplastic □ Antiviral □ Asthma □ CNS Agents □ Immunosuppressants □ Orphan Drugs □ Other (Please Specify)

14. If there was a particular drug class that you believe requires more information to manage, what would it be?

15. Would you like to have access to a predictive tool for dosing guidance that would let you individualize patient treatment?

□Yes □Maybe □No
